# Supplementary material for: From Fruit Waste to Medical Insight: The Comprehensive Role of Watermelon Rind Extract on Renal Adenocarcinoma Cellular and Transcriptomic Dynamics
Source: Int J Mol Sci. 2023 Oct 26;24(21):15615. doi: 10.3390/ijms242115615 (PMC10647773; doi:10.3390/ijms242115615)
Supplement: Supplementary file 1 [file ijms-24-15615-s001.zip › supplemenarty Table S1.pdf]

GeneID	baseMean	log2FoldChange	lfcSE	pvalue	padj	Regulation	Annotation
NPTX1	72.01937727	3.822151586	0.3692	4.0793E-25	1.40205E-22	UP	neuronal pentraxin 1(NPTX1)
LTB	179.4225109	2.944053843	0.22385	1.6534E-39	1.23681E-36	UP	lymphotoxin beta(LTB)
PECAM1	9.255016716	2.818549768	0.81344	0.0005303	0.00333193	UP	platelet and endothelial cell adhesion molecule 1(PECAM1)
HMOX1	5015.831477	2.791739716	0.42932	7.8896E-11	3.72984E-09	UP	heme oxygenase 1(HMOX1)
VNN1	11.21702294	2.722067892	0.73444	0.00021028	0.001563837	UP	vanin 1(VNN1)
ENSG00000289088	9.23435556	2.579239162	0.83977	0.00213085	0.010593432	UP	#N/A
TRIM31	9.080422633	2.578310857	0.87069	0.00306423	0.014206294	UP	tripartite motif containing 31(TRIM31)
CXCL8	391.2561874	2.518431015	0.1521	1.399E-61	2.96515E-58	UP	C-X-C motif chemokine ligand 8(CXCL8)
CXCL2	59.46263547	2.390913463	0.3307	4.8345E-13	3.41557E-11	UP	C-X-C motif chemokine ligand 2(CXCL2)
ENSG00000217825	9.987785824	2.351695319	0.73439	0.00136368	0.007357624	UP	#N/A
CSF2	191.4758711	2.293901374	0.22488	1.9688E-24	6.25941E-22	UP	colony stimulating factor 2(CSF2)
OR2I1P	125.0513163	2.231299376	0.23457	1.8641E-21	4.64817E-19	UP	olfactory receptor family 2 subfamily I member 1 pseudogene(OR2I1P)
FBXO2	11.32453863	2.109406261	0.70507	0.00277372	0.013147017	UP	F-box protein 2(FBXO2)
TRAF1	28.9752565	2.107063383	0.47684	9.9249E-06	0.000117083	UP	TNF receptor associated factor 1(TRAF1)
ENSG00000234537	14.6244859	2.056621595	0.66044	0.00184553	0.009421767	UP	#N/A
FAM138E	19.25950678	2.045475997	0.53972	0.00015074	0.001184009	UP	family with sequence similarity 138 member E(FAM138E)
MIR3142HG	25.619447	2.011663727	0.4538	9.2965E-06	0.000110283	UP	MIR3142 host gene(MIR3142HG)
ANO9	52.17638965	1.933822361	0.33134	5.333E-09	1.6501E-07	UP	anoctamin 9(ANO9)
NAGLU	12.47450292	1.921874255	0.66472	0.00383688	0.01715669	UP	N-acetyl-alpha-glucosaminidase(NAGLU)
ZMIZ1-AS1	13.95999556	1.903714488	0.67565	0.0048386	0.020724991	UP	ZMIZ1 antisense RNA 1(ZMIZ1-AS1)
ARL2BP	13.29894498	1.896221499	0.66549	0.00438084	0.019118456	UP	ADP ribosylation factor like GTPase 2 binding protein(ARL2BP)
KDF1	9.1253128	1.864605173	0.75997	0.01414606	0.049178644	UP	keratinocyte differentiation factor 1(KDF1)
SLC6A12	9.880739992	1.862599952	0.72337	0.01002726	0.03734017	UP	solute carrier family 6 member 12(SLC6A12)
COL17A1	15.60890675	1.839391497	0.59518	0.00199846	0.010065131	UP	collagen type XVII alpha 1 chain(COL17A1)
CITED4	560.0368999	1.796919444	0.11852	6.3472E-52	7.33794E-49	UP	Cbp/p300 interacting transactivator with Glu/Asp rich carboxy-terminal domain 4(CITED4)
C3	494.7153689	1.788111029	0.12647	2.2122E-45	2.16402E-42	UP	complement C3(C3)
ALPK3	9.562138286	1.786908455	0.72977	0.01434153	0.049695147	UP	alpha kinase 3(ALPK3)
TNFAIP3	396.8354414	1.77972605	0.16155	3.1798E-28	1.3479E-25	UP	TNF alpha induced protein 3(TNFAIP3)
BIRC3	623.0128444	1.77722347	0.10906	1.0445E-59	1.47594E-56	UP	baculoviral IAP repeat containing 3(BIRC3)
CBLN3	17.21117487	1.768674376	0.56806	0.00184862	0.009433762	UP	cerebellin 3 precursor(CBLN3)
EFEMP2	10.8345093	1.753814923	0.70469	0.01281816	0.045380994	UP	EGF containing fibulin extracellular matrix protein 2(EFEMP2)
CLEC4E	21.97919912	1.729068492	0.47775	0.00029554	0.002074186	UP	C-type lectin domain family 4 member E(CLEC4E)
ENSG00000272141	25.14557013	1.700340866	0.44134	0.00011682	0.000961524	UP	#N/A
GPR156	14.57353297	1.695993966	0.60881	0.0053407	0.022429883	UP	G protein-coupled receptor 156(GPR156)
CTSS	17.74873139	1.650039926	0.53081	0.00188018	0.009556444	UP	cathepsin S(CTSS)
VCAM1	19.38402297	1.627094205	0.51745	0.00166415	0.008648537	UP	vascular cell adhesion molecule 1(VCAM1)
LAMB3	303.2636745	1.612136661	0.15416	1.357E-25	4.79374E-23	UP	laminin subunit beta 3(LAMB3)
LINC00887	11.97109581	1.604240482	0.64463	0.01282322	0.045386292	UP	long intergenic non-protein coding RNA 887(LINC00887)

LINC01772	15.69339334	1.591827341	0.60101	0.00808337	0.031388652	UP	long intergenic non-protein coding RNA 1772(LINC01772)
C2CD4A	25.11483276	1.577962591	0.51937	0.00237966	0.011568106	UP	C2 calcium dependent domain containing 4A(C2CD4A)
ENSG00000229672	29.70834436	1.563669784	0.40503	0.00011308	0.000934986	UP	#N/A
ARL14EPL	33.44418094	1.547690482	0.41506	0.00019239	0.001456353	UP	ADP ribosylation factor like GTPase 14 effector protein like(ARL14EPL)
CXCL3	14.57097679	1.519626104	0.57373	0.00808053	0.031388652	UP	C-X-C motif chemokine ligand 3(CXCL3)
DPP3-DT	16.3276951	1.510365397	0.54382	0.00548124	0.022884074	UP	DPP3 divergent transcript(DPP3-DT)
C1R	15.6821735	1.500354706	0.55657	0.00702407	0.02805437	UP	complement C1r(C1R)
THBD	33.73003086	1.487504183	0.37832	8.4271E-05	0.000728278	UP	thrombomodulin(THBD)
NFKBID	67.02758427	1.4789834	0.2704	4.511E-08	1.08647E-06	UP	NFKB inhibitor delta(NFKBID)
RASD2	31.0572788	1.478241855	0.4063	0.00027447	0.001948874	UP	RASD family member 2(RASD2)
CCDC28B	42.19107667	1.468454983	0.34525	2.1069E-05	0.000220871	UP	coiled-coil domain containing 28B(CCDC28B)
MAPK13	18.53012483	1.467798471	0.50408	0.00359285	0.016231009	UP	mitogen-activated protein kinase 13(MAPK13)
ENSG00000285744	137.5411721	1.467701975	0.20486	7.8131E-13	5.25709E-11	UP	#N/A
LINC00472	44.33167932	1.467452434	0.33673	1.3128E-05	0.000148121	UP	long intergenic non-protein coding RNA 472(LINC00472)
AGPAT1	15.49631238	1.462735555	0.5708	0.01038859	0.038371088	UP	1-acylglycerol-3-phosphate O-acyltransferase 1(AGPAT1)
APOBEC3G	14.80258467	1.460290422	0.57335	0.01086754	0.039770506	UP	apolipoprotein B mRNA editing enzyme catalytic subunit 3G(APOBEC3G)
IL4I1	45.34921914	1.44733932	0.33278	1.3661E-05	0.000153066	UP	interleukin 4 induced 1(IL4I1)
TUBB2B	15.85319846	1.434770083	0.5715	0.01205553	0.043198149	UP	tubulin beta 2B class IIb(TUBB2B)
ICAM1	320.9656981	1.426350434	0.15845	2.213E-19	4.1387E-17	UP	intercellular adhesion molecule 1(ICAM1)
LINC02672	26.91463457	1.405430517	0.43402	0.00120312	0.006629153	UP	long intergenic non-protein coding RNA 2672(LINC02672)
ENSG00000275741	239.75479	1.385389315	0.16881	2.2717E-16	2.83229E-14	UP	#N/A
WFDC3	21.00301148	1.357704581	0.49365	0.00595386	0.024495402	UP	WAP four-disulfide core domain 3(WFDC3)
AKR1B1	17515.52637	1.356628203	0.06861	5.2183E-87	6.63616E-83	UP	aldo-keto reductase family 1 member B(AKR1B1)
PAQR5	68.0395603	1.355607471	0.27015	5.2201E-07	9.19446E-06	UP	progestin and adipoQ receptor family member 5(PAQR5)
LINC00313	33.48070136	1.32952061	0.38735	0.0005983	0.003663275	UP	long intergenic non-protein coding RNA 313(LINC00313)
ENSG00000277435	18.38584871	1.312388295	0.51814	0.01131277	0.041127655	UP	#N/A
AREG	3885.935229	1.310968358	0.31983	4.1499E-05	0.00039591	UP	amphiregulin(AREG)
MAMDC4	160.2137072	1.304738744	0.19427	1.8683E-11	1.011E-09	UP	MAM domain containing 4(MAMDC4)
AATK	24.53520643	1.298116598	0.46136	0.00489803	0.020909104	UP	apoptosis associated tyrosine kinase(AATK)
ENSG00000280191	62.51512713	1.295940649	0.29101	8.4585E-06	0.000101669	UP	long intergenic non-protein coding RNA 1669(LOC102724354)
COL7A1	1589.03139	1.292376567	0.09047	2.7295E-46	2.89259E-43	UP	collagen type VII alpha 1 chain(COL7A1)
TNFRSF9	160.7469794	1.289730042	0.21071	9.3066E-10	3.43674E-08	UP	TNF receptor superfamily member 9(TNFRSF9)
ENSG00000262050	18.01453394	1.288651592	0.50783	0.01116337	0.040642579	UP	#N/A
RNF144B	81.37883568	1.287668834	0.25517	4.5033E-07	8.06605E-06	UP	ring finger protein 144B(RNF144B)
PC	240.0281931	1.287022568	0.17714	3.7162E-13	2.77996E-11	UP	pyruvate carboxylase(PC)
SLC2A12	35.39208808	1.276481779	0.36956	0.00055213	0.003430142	UP	solute carrier family 2 member 12(SLC2A12)
SMIM14	28.71265187	1.276413543	0.41879	0.00230451	0.011271694	UP	small integral membrane protein 14(SMIM14)
TMEM171	34.54129481	1.267209507	0.38006	0.00085547	0.004981249	UP	transmembrane protein 171(TMEM171)
TBC1D22A-DT	43.41795402	1.259709092	0.3514	0.00033736	0.002311507	UP	TBC1D22A divergent transcript(TBC1D22A-DT)

SGPP2	57.97035381	1.255288602	0.32254	9.9451E-05	0.000839225	UP	sphingosine-1-phosphate phosphatase 2(SGPP2)
LINC01443	24.91874054	1.250441712	0.44612	0.0050644	0.021467981	UP	long intergenic non-protein coding RNA 1443(LINC01443)
ENSG00000279561	19.82625654	1.248345459	0.48469	0.0100079	0.037278982	UP	translation initiation factor IF-2-like(LOC105376060)
SOD2	10639.05133	1.24731498	0.07184	1.5752E-67	6.67748E-64	UP	superoxide dismutase 2(SOD2)
ARRB1	72.12746756	1.244050952	0.28366	1.1562E-05	0.00013318	UP	arrestin beta 1(ARRB1)
SLC9A3R2	287.6469071	1.233274911	0.13951	9.5632E-19	1.6002E-16	UP	#N/A
ANPEP	712.5234949	1.223785982	0.31853	0.00012207	0.000997699	UP	alanyl aminopeptidase, membrane(ANPEP)
ENSG00000274213	20.18554316	1.220605622	0.47701	0.01050168	0.038710102	UP	#N/A
LINC02262	48.68181873	1.218514869	0.31781	0.00012601	0.001023745	UP	long intergenic non-protein coding RNA 2262(LINC02262)
OLFML2B	202.9180548	1.212041835	0.18528	6.0898E-11	2.96722E-09	UP	olfactomedin like 2B(OLFML2B)
ENSG00000289839	50.16342455	1.21067541	0.36616	0.00094492	0.005415296	UP	#N/A
LINC00488	23.61292309	1.209221472	0.48425	0.01252193	0.044556985	UP	long intergenic non-protein coding RNA 488(LINC00488)
RHOV	35.87108487	1.208659398	0.41083	0.00326088	0.015003099	UP	ras homolog family member V(RHOV)
CX3CL1	397.9596196	1.206057947	0.12425	2.8329E-22	7.66507E-20	UP	C-X3-C motif chemokine ligand 1(CX3CL1)
SERPINA1	547.49556	1.205216402	0.12851	6.7107E-21	1.47138E-18	UP	serpin family A member 1(SERPINA1)
ST6GALNAC4	683.5497101	1.200235905	0.10438	1.3353E-30	7.07538E-28	UP	ST6 N-acetylgalactosaminide alpha-2,6-sialyltransferase 4(ST6GALNAC4)
PTOV1	80.87839154	1.190356343	0.24657	1.381E-06	2.12108E-05	UP	PTOV1 extended AT-hook containing adaptor protein(PTOV1)
BMF	54.66259972	1.183513353	0.30181	8.8064E-05	0.000755165	UP	Bcl2 modifying factor(BMF)
RHEBL1	48.11097776	1.17295039	0.31872	0.00023304	0.001703166	UP	RHEB like 1(RHEBL1)
ENSG00000248367	24.55624847	1.169803274	0.44915	0.00920084	0.034917082	UP	uncharacterized LOC124901151(LOC124901151)
TPD52L1	37.02536096	1.162282589	0.36245	0.00134267	0.007265863	UP	TPD52 like 1(TPD52L1)
RAB43P1	41.21318978	1.160233022	0.34781	0.00085034	0.00495818	UP	RAB43 pseudogene 1(RAB43P1)
EPCAM-DT	39.04813643	1.160213098	0.44441	0.00903607	0.034445959	UP	EPCAM divergent transcript(EPCAM-DT)
HES7	23.16161629	1.157004607	0.47192	0.01421865	0.049403985	UP	hes family bHLH transcription factor 7(HES7)
PALM3	47.46779657	1.145887278	0.36243	0.00156873	0.008270951	UP	paralemmin 3(PALM3)
NFKBIA	1195.868859	1.140983535	0.10184	3.8979E-29	1.77033E-26	UP	NFKB inhibitor alpha(NFKBIA)
ENSG00000290762	43.03785211	1.137233063	0.37207	0.00223951	0.01101311	UP	#N/A
NFKB2	2686.280572	1.134197069	0.06842	1.0108E-61	2.57092E-58	UP	nuclear factor kappa B subunit 2(NFKB2)
ENSG00000290644	55.19462761	1.133528543	0.30875	0.00024123	0.001755003	UP	#N/A
RHBDL1	23.59709628	1.127426241	0.443	0.01092899	0.039940739	UP	rhomboid like 1(RHBDL1)
NMRAL2P	26.952878	1.122741835	0.42062	0.00760255	0.029904632	UP	NmrA like redox sensor 2, pseudogene(NMRAL2P)
VEGFC	32.4937423	1.120809677	0.4071	0.00590262	0.024339678	UP	vascular endothelial growth factor C(VEGFC)
C1QL4	36.354995	1.12045879	0.38711	0.00379867	0.017018137	UP	complement C1q like 4(C1QL4)
ENSG00000255050	28.66124763	1.117491658	0.41685	0.00734447	0.029051194	UP	#N/A
CD82	178.4495495	1.109222868	0.23553	2.4843E-06	3.56575E-05	UP	CD82 molecule(CD82)
CLIP4	48.60500716	1.105718912	0.33001	0.00080656	0.004748612	UP	CAP-Gly domain containing linker protein family member 4(CLIP4)
TMEM156	26.30152032	1.102133461	0.42546	0.00958441	0.036039312	UP	transmembrane protein 156(TMEM156)
UNKL	536.7160737	1.101863646	0.11785	8.8131E-21	1.89959E-18	UP	unk like zinc finger(UNKL)
ZFYVE28	150.6587378	1.099336689	0.20633	9.9219E-08	2.18678E-06	UP	zinc finger FYVE-type containing 28(ZFYVE28)
ENSG00000253507	24.1692893	1.099080346	0.43576	0.01166163	0.042107041	UP	#N/A

NFE2L3	320.5436574	1.09901105	0.14859	1.3987E-13	1.14759E-11	UP	NFE2 like bZIP transcription factor 3(NFE2L3)
TRIM16L	1775.629311	1.088105329	0.07852	1.1441E-43	9.69946E-41	UP	tripartite motif containing 16 like(TRIM16L)
CCDC200	27.43022098	1.075686636	0.40965	0.00864229	0.033223685	UP	coiled-coil domain containing 200(CCDC200)
LIPG	57.06281364	1.075590149	0.28398	0.00015213	0.001193288	UP	lipase G, endothelial type(LIPG)
S100A4	215.4142698	1.074798386	0.16967	2.3785E-10	1.01164E-08	UP	S100 calcium binding protein A4(S100A4)
TRANK1	130.0570278	1.071438154	0.20342	1.385E-07	2.9233E-06	UP	tetratricopeptide repeat and ankyrin repeat containing 1(TRANK1)
MEGF6	3068.428196	1.068206684	0.06474	3.7433E-61	6.80056E-58	UP	multiple EGF like domains 6(MEGF6)
NFKBIE	555.4858127	1.065145553	0.12561	2.2551E-17	3.25882E-15	UP	NFKB inhibitor epsilon(NFKBIE)
TMEM158	186.7110155	1.061667088	0.19642	6.4825E-08	1.49615E-06	UP	transmembrane protein 158(TMEM158)
SPACA6	55.72906649	1.060982575	0.29573	0.00033369	0.002291319	UP	sperm acrosome associated 6(SPACA6)
IQCD	54.6735817	1.0590693	0.30008	0.00041676	0.002731955	UP	IQ motif containing D(IQCD)
ZC3H12A	504.4137214	1.058928366	0.11519	3.8225E-20	7.96899E-18	UP	zinc finger CCCH-type containing 12A(ZC3H12A)
HSF4	56.45796623	1.058853794	0.32315	0.00105045	0.005924004	UP	heat shock transcription factor 4(HSF4)
AKR1C2	649.6438404	1.058573734	0.1053	8.9069E-24	2.63416E-21	UP	aldo-keto reductase family 1 member C2(AKR1C2)
GGT4P	948.2916375	1.0578441	0.11213	3.9495E-21	9.13198E-19	UP	gamma-glutamyltransferase 4 pseudogene(GGT4P)
ENSG00000259354	37.81678704	1.057276601	0.38407	0.00590845	0.024355831	UP	#N/A
ADAMTS7	366.8771864	1.047212012	0.12953	6.2416E-16	7.15087E-14	UP	ADAM metallopeptidase with thrombospondin type 1 motif 7(ADAMTS7)
ENSG00000236938	39.34367145	1.04604922	0.36438	0.00409468	0.018105699	UP	#N/A
GRAMD2A	32.17367262	1.045240244	0.37401	0.00519536	0.021928126	UP	GRAM domain containing 2A(GRAMD2A)
TPCN1	4273.786373	1.044200672	0.06031	3.6169E-67	1.14991E-63	UP	two pore segment channel 1(TPCN1)
PAX8	114.0774797	1.04008372	0.20295	2.9801E-07	5.63949E-06	UP	paired box 8(PAX8)
BRICD5	28.12074621	1.039299423	0.40392	0.01008029	0.037515111	UP	BRICHOS domain containing 5(BRICD5)
SLCO4A1	3566.061844	1.031726853	0.06606	5.4211E-55	6.89398E-52	UP	solute carrier organic anion transporter family member 4A1(SLCO4A1)
KCNK3	1180.556968	1.026865059	0.11389	1.9414E-19	3.68494E-17	UP	potassium two pore domain channel subfamily K member 3(KCNK3)
ZNF837	43.9438911	1.026531985	0.32915	0.00181631	0.009309964	UP	zinc finger protein 837(ZNF837)
PGGHG	469.639066	1.026377088	0.10877	3.863E-21	9.09732E-19	UP	protein-glucosylgalactosylhydroxylysine glucosidase(PGGHG)
COL6A1	1696.831616	1.024081986	0.08387	2.7133E-34	1.72527E-31	UP	collagen type VI alpha 1 chain(COL6A1)
BFSP1	103.4601228	1.016356666	0.23302	1.2902E-05	0.000145976	UP	beaded filament structural protein 1(BFSP1)
ZNF467	304.5514326	1.013726249	0.35042	0.00381712	0.017080335	UP	zinc finger protein 467(ZNF467)
LACTB	59.9325034	1.007908666	0.30123	0.00081999	0.004812089	UP	lactamase beta(LACTB)
AKR1C1	2781.012441	1.007484428	0.07626	7.5294E-40	5.98445E-37	UP	aldo-keto reductase family 1 member C1(AKR1C1)
ATG16L2	131.5606598	1.001999864	0.20338	8.364E-07	1.37778E-05	UP	autophagy related 16 like 2(ATG16L2)
CELF2	68.96008457	-1.012165448	0.26166	0.00010963	0.00091243	DOWN	CUGBP Elav-like family member 2(CELF2)
KCNH1	40.57583995	-1.01380309	0.35816	0.00464638	0.020077479	DOWN	potassium voltage-gated channel subfamily H member 1(KCNH1)
CDH6	631.9834499	-1.044599459	0.14216	2.0085E-13	1.57671E-11	DOWN	cadherin 6(CDH6)
SMIM24	80.05265014	-1.044849866	0.26378	7.4607E-05	0.000652979	DOWN	small integral membrane protein 24(SMIM24)
DOCK2	660.634138	-1.058742034	0.1035	1.4606E-24	4.76266E-22	DOWN	dedicator of cytokinesis 2(DOCK2)
PAX8-AS1	176.2260243	-1.058885169	0.17219	7.7741E-10	2.95114E-08	DOWN	PAX8 antisense RNA 1(PAX8-AS1)
ENSG00000242553	51.77259918	-1.063421863	0.31092	0.0006256	0.003808379	DOWN	#N/A

ANKRD1	466.1436283	-1.080370398	0.16656	8.7844E-11	4.09198E-09	DOWN	ankyrin repeat domain 1(ANKRD1)
TAF1A-AS1	27.34881464	-1.086638886	0.42359	0.01030917	0.038122062	DOWN	TAF1A antisense RNA 1(TAF1A-AS1)
CCDC80	25.54908672	-1.0890936	0.42049	0.00959569	0.036060384	DOWN	coiled-coil domain containing 80(CCDC80)
RHOU	28.53912737	-1.10777548	0.45112	0.0140649	0.048963406	DOWN	ras homolog family member U(RHOU)
SLC16A9	34.68302167	-1.1715508	0.38125	0.00211951	0.010549451	DOWN	solute carrier family 16 member 9(SLC16A9)
ENC1	987.336713	-1.17558341	0.26977	1.3138E-05	0.000148121	DOWN	ectodermal-neural cortex 1(ENC1)
GREB1	23.66531699	-1.200291699	0.46904	0.01049563	0.038699026	DOWN	growth regulating estrogen receptor binding 1(GREB1)
NEFL	1302.998274	-1.200493071	0.41879	0.00414945	0.01827801	DOWN	neurofilament light chain(NEFL)
HRK	27.53909215	-1.29957579	0.43666	0.0029187	0.013691315	DOWN	harakiri, BCL2 interacting protein(HRK)
PKHD1	63.9089557	-1.311859039	0.28324	3.6288E-06	4.96206E-05	DOWN	PKHD1 ciliary IPT domain containing fibrocystin/polyductin(PKHD1)
HORMAD2-AS1	16.38130256	-1.375725708	0.5453	0.0116396	0.042051375	DOWN	HORMAD2 and MTMR3 antisense RNA 1(HORMAD2-AS1)
ENSG00000286856	71.76167158	-1.38967043	0.27463	4.1884E-07	7.54447E-06	DOWN	#N/A
UNC79	16.10605975	-1.401693747	0.56802	0.01359967	0.047539019	DOWN	unc-79 homolog, NALCN channel complex subunit(UNC79)
ENSG00000248199	37.08015659	-1.404986826	0.4001	0.0004455	0.00287586	DOWN	#N/A
FGB	1322.570851	-1.441683784	0.08832	6.7895E-60	1.07928E-56	DOWN	fibrinogen beta chain(FGB)
SLCO4C1	57.80931232	-1.500449851	0.31815	2.4032E-06	3.46892E-05	DOWN	solute carrier organic anion transporter family member 4C1(SLCO4C1)
NPNT	90.44313417	-1.529088042	0.23791	1.299E-10	5.81654E-09	DOWN	nephronectin(NPNT)
ARHGAP28	56.51796503	-1.53301957	0.30112	3.5619E-07	6.57424E-06	DOWN	Rho GTPase activating protein 28(ARHGAP28)
C1orf116	12.83673795	-1.56932167	0.63039	0.01279389	0.045320324	DOWN	chromosome 1 open reading frame 116(C1orf116)
ENSG00000276851	37.66641036	-1.623060278	0.40115	5.2107E-05	0.000480181	DOWN	#N/A
APBA1	77.73145949	-1.734613144	0.27467	2.697E-10	1.13192E-08	DOWN	amyloid beta precursor protein binding family A member 1(APBA1)
ENSG00000288085	20.91837028	-1.778851101	0.52033	0.00062929	0.003823531	DOWN	#N/A
SLAMF7	13.36894147	-1.840054142	0.67062	0.00607324	0.024930087	DOWN	SLAM family member 7(SLAMF7)
NKAIN4	10.63048692	-1.847616417	0.73287	0.01169974	0.042196702	DOWN	sodium/potassium transporting ATPase interacting 4(NKAIN4)
LINC00571	10.80096799	-1.855056546	0.75572	0.01410018	0.049059366	DOWN	long intergenic non-protein coding RNA 571(LINC00571)
UNC13C	8.868999783	-1.944259269	0.75296	0.00981861	0.03671916	DOWN	unc-13 homolog C(UNC13C)
EPHA7	829.8016104	-2.087931878	0.10708	1.1403E-84	7.25051E-81	DOWN	EPH receptor A7(EPHA7)
SLC26A5-AS1	9.85255604	-2.285084696	0.74163	0.00206193	0.010311261	DOWN	SLC26A5 antisense RNA 1(SLC26A5-AS1)
SULT1B1	23.11920729	-2.333807748	0.49817	2.8029E-06	3.9407E-05	DOWN	sulfotransferase family 1B member 1(SULT1B1)
FLT3	14.35560724	-2.335935596	0.62732	0.00019634	0.001480949	DOWN	fms related receptor tyrosine kinase 3(FLT3)
