# Supplementary material for: From Fruit Waste to Medical Insight: The Comprehensive Role of Watermelon Rind Extract on Renal Adenocarcinoma Cellular and Transcriptomic Dynamics
Source: Int J Mol Sci. 2023 Oct 26;24(21):15615. doi: 10.3390/ijms242115615 (PMC10647773; doi:10.3390/ijms242115615)
Supplement: Supplementary file 1 [file ijms-24-15615-s001.zip › Supplementary Figure S1.pdf]

A

B

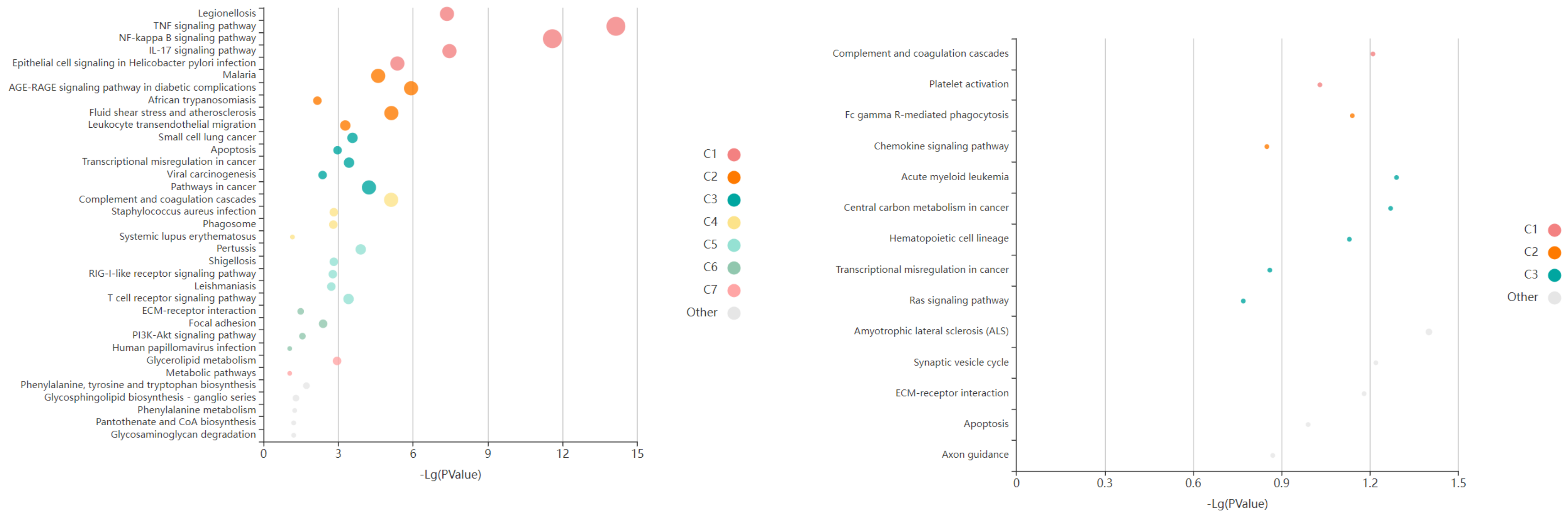

Supplementary Figure S1 Scatter plot of top 20 enriched KEGG pathways among upregulated (**A**) and downregulated (**B**). DEGs in renal cell adenocarcinoma against WRE treatment **(44.8 mg)**). The rich factor is the ratio of the number of DEGs to total gene number in a pathway. The color and size of the dots represent the range of p-values and the number of DEGs mapped to the indicated pathways, Respectively.
