# Supplementary material for: From Fruit Waste to Medical Insight: The Comprehensive Role of Watermelon Rind Extract on Renal Adenocarcinoma Cellular and Transcriptomic Dynamics
Source: Int J Mol Sci. 2023 Oct 26;24(21):15615. doi: 10.3390/ijms242115615 (PMC10647773; doi:10.3390/ijms242115615)
Supplement: Supplementary file 1 [file ijms-24-15615-s001.zip › Supplementary Figure S2.pdf]

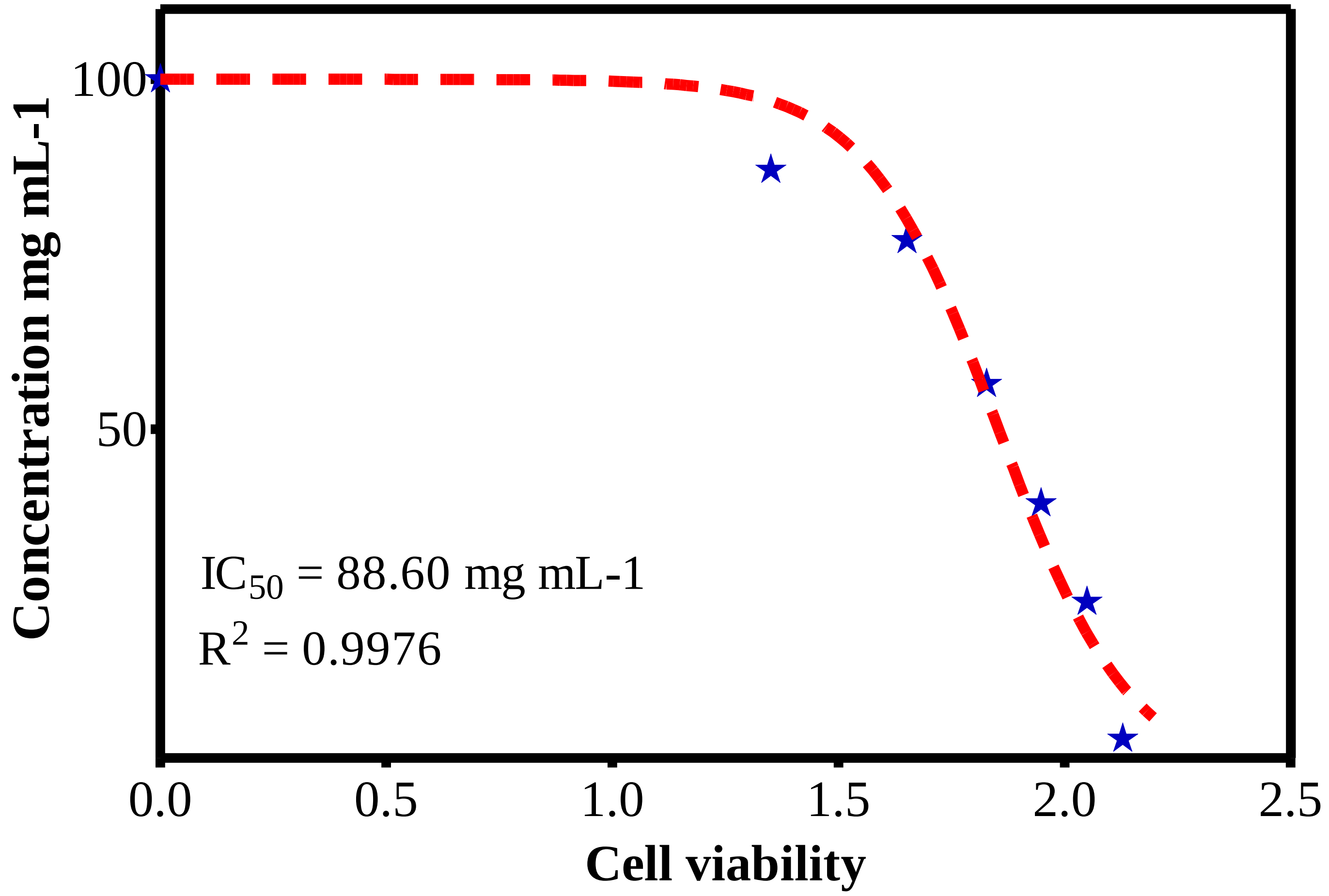


**Supplementary Figure S2** The effect of different concentrations of *Citrullus lanatus rind* extracts and IC50 Value of human renal adenocarcinoma cells viability. X-axis indicating the cell viability and Y-axis indicating Concentration. The cell viability was assessed after 24 h by WST-8 assay.
